# Sepsis is an important healthcare burden in Latin America: a call to action!

**DOI:** 10.5935/0103-507X.20180061

**Published:** 2018

**Authors:** Luciano Cesar Pontes Azevedo, Alexandre Biasi Cavalcanti, Thiago Lisboa, Felipe Dal Pizzol, Flavia Ribeiro Machado

**Affiliations:** 1 Instituto Latino Americano de Sepse - São Paulo (SP), Brasil.

Sepsis is a life-threatening organ dysfunction caused by a dysregulated host response to infection. The precise incidence of sepsis is unknown and there is a lack of populational studies on the disease, especially from low and middle-income countries. However, extrapolations of populational data from high-income countries suggest a number of 30 million sepsis cases annually worldwide with approximately 6 million deaths.^(1)^ In Latin-American (LATAM) countries, the few available studies also suggest that sepsis represents a significant healthcare issue. A previous study from Brazil demonstrated that infection-associated organ dysfunction was related to up to 22% of all deaths in the country in 2010.^(2)^ In Brazilian intensive care units (ICUs), sepsis prevalence is 30% and hospital mortality rate for ICU patients is 55%.^(3)^ Studies from Colombia and Argentina also reported mortality rates for septic shock ranging from 45.6% to 51%.^(4,5)^ Reasons for this significant burden of sepsis may include areas with inadequate provision of clean water, sanitation and nutrition, inadequate vaccination, as well as reduced awareness of sepsis among lay people and healthcare personnel, low access to intensive care services and an increased incidence of healthcare-associated infections.^(6)^

In order to tackle the burden of sepsis worldwide, in 2012 the Global Sepsis Alliance (GSA) created the first World Sepsis Day (WSD) as a launch platform for the World Sepsis Declaration. The purposes of WSD are to increase the perception of sepsis importance among lay public, healthcare workers and policy makers and to encourage quality improvement programs for sepsis early identification and treatment. Since then, initiatives to improve sepsis awareness have increased worldwide with events for lay public, healthcare staff and policy makers. These initiatives have culminated with the approval on May 26^th^, 2017 of a sepsis resolution (WHA70.7) by the World Health Organization (WHO).^(7)^ The WHO resolution recognizes sepsis as a major threat to patient safety and global health and urges the member states to start initiatives to improve sepsis prevention, recognition and treatment. This resolution has the potential to save millions of lives, however for that happening it requires coordinated efforts by politicians, policymakers, health care administrators, researchers, and clinicians working in all health care settings.^(8)^

Also, regional alliances may be important in the sepsis agenda in order to foster collaboration between countries with similarities and improve coordinated regional advocacy as compared to single countries alone. As such, in May 30^th^ 2018, the *Instituto Latino Americano de Sepse* (ILAS) organized a meeting for representatives of LATAM countries. This meeting was supported by the Pan America Health Organization (PAHO) and had the purpose of identifying common problems and discussing possible approaches for sepsis agenda on LATAM countries in accordance with the WHO resolution. Representatives from the Global Sepsis Alliance and Pan America Health Organization were present, as well as delegates from Argentina, Brazil, Chile, Colombia, Mexico, Peru, and Uruguay. A representative of the *Consorcio Centroamericano y Del Caribe de Terapia Intensiva* (COCECATI) was also present representing the following countries: Belize, Guatemala, El Salvador, Costa Rica, Nicaragua, Panama, Dominican Republic, Puerto Rico and Cuba. In this meeting, the "São Paulo Declaration" (Appendix 1) was approved. The declaration contains the main requests from the group for government authorities, policy makers, healthcare managers and professionals, and associated societies to support national and international commitments to improve the prevention, diagnosis, and treatment of sepsis and to dedicate human and financial resources to these goals. It is available to be downloaded and signed at ILAS website (www.ilas.org.br). We request the readers to sign and spread the "São Paulo Declaration" so that ILAS mission can be fully accomplished: "A continent with no more avoidable sepsis deaths".

## Appendix 1 São Paulo Declaration.

**São Paulo Declaration**

**Sepsis - the Major Cause of Preventable Death and Disability in Latin America**

**A Call for Action to Reduce the Burden of Sepsis**

Sepsis is a major cause of preventable deaths in Latin America (LATAM) countries and is the most common cause of death from infection.

During the Latin-American Sepsis Institute meeting in São Paulo, Brazil, on the 30^th^ of May 2018, delegates representing 16 LATAM countries called for urgent action by governments, healthcare workers and the community to support national and international commitments to improve the prevention, diagnosis, and treatment of sepsis and to dedicate human and financial resources to these goals. The delegates supported the following declaration:

**Noting** that sepsis is recognized as a global health priority by WHO Resolution WHA A70/13 of 2017 and that member nations are urged to adopt national policies to improve the prevention, recognition and treatment of sepsis;

**Recognizing** that despite the unacceptable number of deaths and disabilities caused by sepsis, awareness of sepsis among healthcare providers and lay public in LATAM countries is very low;

**Stressing** that there is wide variation among LATAM settings regarding healthcare services to treat sepsis;

**Identifying** that hospital-acquired infections and antimicrobial resistance are a major healthcare issue in LATAM countries;

We urge government authorities, policy makers, healthcare managers, professionals, universities and associated societies to:

- endorse the WHO Resolution on Sepsis and establish national action plans to prevent sepsis, to enhance early recognition and management in a continuous effort to improve access to care and adequate resources and to reduce ineqality,
- focus on sepsis prevention by providing adequate sanitation, vaccination to at-risk groups and adequate nutrition, as well as reducing maternal and pediatric deaths,
- cooperate in partnership to ensure adequate sepsis treatment in all nations, through undergraduate and post graduate training of healthcare professionals focused on improving outcomes in both patients and survivors, recognizing that the establishment of adequate national policies to treat sepsis in one country will clearly benefit other nations,
- promote sepsis awareness among lay people and healthcare workers including recognizing World Sepsis Day (September 13^th^) as a national date,
- implement measures aimed at minimizing the risk of the development and spread of antimicrobial resistance and hospital-acquired infections,
- promote collaborative research to further understand the burden of sepsis as well as to identify local perspectives and priorities for adequate recognition and treatment of sepsis.

São Paulo, May 30^th^, 2018.

## References

[1] Kissoon N, Reinhart K, Daniels R, Machado MF, Schachter RD, Finfer S (2017). Sepsis in children: global implications of the World Health
Assembly Resolution on Sepsis. Pediatr Crit Care Med.

[2] Taniguchi LU, Bierrenbach AL, Toscano CM, Schettino GP, Azevedo LC (2014). Sepsis-related deaths in Brazil: an analysis of the national
mortality registry from 2002 to 2010. Crit Care.

[3] Machado FR, Cavalcanti AB, Bozza FA, Ferreira EM, Angotti Carrara FS, Sousa JL, Caixeta N, Salomao R, Angus DC, Pontes Azevedo LC, SPREAD Investigators, Latin American Sepsis Institute Network (2017). The epidemiology of sepsis in Brazilian intensive care units (the
Sepsis PREvalence Assessment Database, SPREAD): an observational
study. Lancet Infect Dis.

[4] Rodríguez F, Barrera L, De La Rosa G, Dennis R, Dueñas C, Granados M (2011). The epidemiology of sepsis in Colombia: a prospective multicenter
cohort study in ten university hospitals. Crit Care Med.

[5] Estenssoro E, Kanoore Edul VS, Loudet CI, Osatnik J, Ríos FG, Vázquez DN, Pozo MO, Lattanzio B, Pálizas F, Klein F, Piezny D, Rubatto Birri PN, Tuhay G, Díaz A, Santamaría A, Zakalik G, Dubin A, SATISEPSIS Investigators (2018). Predictive validity of Sepsis-3 definitions and sepsis outcomes
in critically ill patients: a cohort study in 49 ICUs in
Argentina. Crit Care Med.

[6] Machado FR, Azevedo LC (2018). Sepsis: a threat that needs a global solution. Crit Care Med.

[7] World Health Organization (WHO) (c2018). Sepsis. Improving the prevention, diagnosis and clinical managment of
sepsis.

[8] Reinhart K, Daniels R, Kissoon N, Machado FR, Schachter RD, Finfer S (2017). Recognizing sepsis as a global health priority - A WHO
resolution. N Engl J Med.

